# The Metabolism of Salidroside to Its Aglycone *p*-Tyrosol in Rats following the Administration of Salidroside

**DOI:** 10.1371/journal.pone.0103648

**Published:** 2014-08-07

**Authors:** Na Guo, Meixuan Zhu, Xuejiao Han, Dan Sui, Yang Wang, Qian Yang

**Affiliations:** 1 School of Municipal and Environmental Engineering, Harbin Institute of Technology, Harbin, China; 2 Alkali Soil Natural Environmental Science Center, Northeast Forestry University; Key Laboratory of Saline-alkali Vegetation Ecology Restoration in Oil Field, Ministry of Education, Harbin, China; 3 School of Life Science and Technology, Harbin Institute of Technology, Harbin, China; 4 Management Office of Laboratory and Equipment (Center of Analysis and Testing), Northeast Forestry University, Harbin, China; Universidade Federal do Rio de Janeiro, Brazil

## Abstract

Salidroside is one of the major phenolic glycosides in *Rhodiola*, which has been reported to possess various biological activities. In the present study the *in vivo* deglycosylation metabolism of salidroside was investigated and its aglycone *p*-tyrosol but not the original salidroside was identified as the main form in rat tissues following the administration of salidroside. After the i.v. administration of salidroside at a dose of 50 mg/kg in rats, salidroside was quantified only in the liver, kidney and heart tissues. The highest level of *p*-tyrosol was detected in the heart, followed by the spleen, kidney, liver and lungs, in order. Salidroside was detected only in the liver, in contrast, *p-*tyrosol was detectable in most tissues except the brain, and the kidney tissues contained a significant amount of *p-*tyrosol compared to the other tissues after the i.g. administration of 100 mg/kg salidroside. The excretion behaviour revealed that the administrated salidroside mainly eliminated in the form of salidroside but not its aglycone metabolite *p*-tyrosol through urine. After i.v. and i.g. administration in rats, 64.00% and 23.80% of the total dose was excreted through urine in the form of salidroside, respectively. In addition, 0.19% and 2.25% of the dose was excreted in the form of *p-*tyrosol through urine after i.v. and i.g. administration, respectively. The faecal salidroside and *p*-tyrosol concentrations were 0.3% and 1.48% of the total dose after i.v. administration, respectively. After the i.g. administration of salidroside, trace salidroside and *p*-tyrosol were quantified in faeces within 72 h. In addition, the biliary excretion levels of salidroside after i.v. and i.g. administration were 2.86% and 0.02% of the dose, respectively. The obtained results show that salidroside was extensively metabolised to its aglycone *p*-tyrosol and distributed to various organs and the orginal salidroside was cleared rapidly through urine following the administration of salidroside.

## Introduction

Phenol glycosides are produced by most fruits, vegetables and herbal medicines. As a result, most research on their metabolism has been performed after oral administration. In the last century, it was commonly accepted that polyphenol glycosides remained intact until they reached the colon, where they underwent deglycosylation by colonic microflora, releasing aglycones [1]. However, some flavonols and isoflavones appear in the plasma within 30 min of ingestion, indicating rapid absorption in the small intestine [1]. Deglycosylation by small intestinal epithelial cell β-glucosidases is a critical step in the absorption and metabolism of dietary phenol glycoside [2].

**Figure 1:**
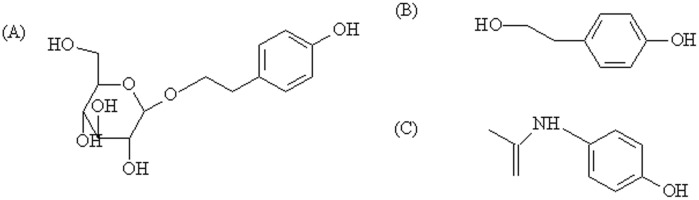
Chemical structures of (A) salidroside, (B) *p-*tyrosol and (C) paracetamol (IS).

Salidroside, *p*-hydroxyphenylethyl-*O*-*β*-D-glucopyranoside (structure is shown in **Figure 1A**), is one of the major phenolic glycosides in *Rhodiola*, often used as one of the criteria to evaluate the quality of *Rhodiola* [3]. It possesses various biological activities, including antifatigue [4], anticancer [5], antioxidant [6], anti-aging, antiviral [7], hepatoprotective effect [8], protective effects against cardiomyocyte death and anoxia/reoxygenation damages on myocardium [9], [10]. *p-*Tyrosol, 2-(4-hydroxyphenyl)ethanol (aglycone of salidroside, **Figure 1B**), is an abundant bio-phenol in extra virgin olive oil [11], which has been shown to protect Caco-2 cells against oxidised LDL-induced oxidative cellular damage and cell retraction caused by oxidised LDL [12]. Vivancos M *et al* recently reported that *p*-tyrosol treatment reduced oxidative stress stimulated by oxidised LDL in RAW 264.7 macrophages [13]. Plotnikov *et al* reported that intra-gastric *p*-tyrosol treatment in male Wistar rats led to a pronounced reduction in platelet aggregation and blood viscosity in these rats [14]. Additionally, it has been shown that the intravenous administration of *p*-tyrosol 10 min prior to coronary occlusion in an *in vivo* acute myocardial ischemia model of Wistar rats significantly reduced the arrhythmic activity occurring during myocardial ischemia and reperfusion [15]. Moreover, *p*-tyrosol induced myocardial protection against ischemia-induced stress, thereby prompting the development of a new drug to combat ischemic heart diseases (IHD) while revealing potential therapeutic molecular targets such as FOXO3a and SIRT1 that can be modulated to precondition the heart to overcome ischemic stress and to inhibit the activity of leukocyte 5-lipoxygenase [16].

During the past decade, *Rhodiola* extract, which includes salidroside as one of its standard active components, has been valued as a strengthening tonic to increase physical and mental stamina, marketed under several brands and sold on major websites (including amazon.com, buy.com, and drugstore) and drug stores (Walgreens and GNC) in the United States. Healthy bread containing *Rhodiola* as a functional ingredient was developed by mixing *Rhodiola* extracts with wheat flour, which showed higher water holding capacity and longer shelf life, as well as a unique flavour and good taste [17]. In addition, it has been used to prepare whitening and anti-aging cosmetics [18].

*p*-Tyrosol was identified as the deglycosylated metabolite of salidroside in rat plasma after intravenous (i.v.) administration at a dose of 50 mg/kg, but it was not detectable after intragastric gavage (i.g. 100 mg/kg) administration through HPLC-photodiode array (PDA) detection and LC-MS/MS analysis in our previous study. The results accounted for approximately 2% of the salidroside as its aglycone metabolite, *p*-tyrosol, in the plasma after i.v. administration [19]. According to the literature, deglycosylation metabolism is expected to occur after the i.g. administration of salidroside other than after i.v. administration. Thus, we sought to determine whether deglycosylation metabolism also occurred following i.g. administration.

Tissue distribution studies are essential to understand the distribution and accumulation of a compound and its metabolites, especially in relation to potential sites of action; this information may be useful for designing toxicology and pharmacology studies and for interpreting the results of these experiments [20]. Excretion studies are a significant approach to the investigation of drug elimination *in vivo*. Drugs can be excreted from the body through excretory organs by prototype or their metabolites through urine, bile and faeces. Paramount to the development of a drug for certain disease treatment is understanding its distribution, metabolism and excretion after oral administration in preclinical species [21]. Due to the lack of studies regarding the tissue distribution and excretion of salidroside in rats, the present study aims to quantify salidroside and *p*-tyrosol in rat tissues and excretory samples and to determine whether deglycosylation occurs following i.g. administration, as well as to compare and elucidate the tissue distribution and excretion of salidroside and its metabolite *p*-tyrosol in rats after i.v. and i.g. administration of salidroside, to better understand the *in vivo* deglycosylation metabolism of salidroside.

## Materials and Methods

### Chemicals

Salidroside (CAS number 10338-51-9) was purchased from the National Institutes for Food and Drug Control (Beijing, China). Paracetamol (CAS number 103-90-2), the internal standard (IS), was purchased from Sinopharm Chemical Reagent Co. Ltd (Shanghai, China). *p-*Tyrosol (CAS number 501-94-0) and HPLC-grade acetonitrile were obtained from Sigma (St. Louis, MO, USA). Ultra-pure water was produced by a Millipore Milli-Q system (Billerica, MA, USA). All other reagents and solvents used were commercially available and of reagent grade.

### Ethics Statement

Animal handling procedures were according to standard operating procedures approved by the institutional animal care and use committee at Northeast Forestry University. All efforts were made to minimize suffering and the procedure was performed under sodium pentobarbital anesthesia if necessary.

### Animals

Healthy male Wistar rats (ICR, 200±20 g) were obtained from the Laboratory Animal Center of Jilin University (Changchun, Jilin Province, China). Rats were housed under specific pathogen-free conditions with 12∶12 light: dark cycle at 22±2°C and 60±5% humidity. Sterile water and chow were available ad libitum.

### Tissue sample collections

Thirty-five healthy male rats were randomly assigned to 7 groups, and each group contained 5 rats. Animals in the 7 groups were administered salidroside by i.v. (12.5 mg/mL in saline) at a dose of 50 mg per kg body weight through the vena caudalis (i.v. 50 mg/kg). In another group of thirty-five healthy male rats, each rat received an i.g. administration of 100 mg/kg (12.5 mg/mL salidroside in saline). The rats were euthanised by decapitation before and at 0.17, 0.33, 0.5, 1.0, 2.0, 4.0 h after administration. The tissue samples were weighed rapidly and immersed in normal saline solution, blotted on filter paper, weighed to determine the fresh weight, and homogenised in phosphate buffer solution (pH 7.4) (50% tissue w/v). The obtained tissue homogenates were stored at −20°C until analysis, which was performed using the procedure described below.

### Collections of urine, faeces and bile

For urinary and faecal excretion experiments, 5 rats were administered salidroside at a single dose of 50 mg·kg^−1^ via i.v. or at a dose of 100 mg/kg via i.g., as described above. The rats were then placed in stainless-steel metabolic cages, one rat per cage. Urine and faeces were collected separately from each rat at intervals of −12∼0 h predose and 0∼12, 12∼24, 24∼48 and 48∼72 h postdose. The volume of each urinary sample was recorded, and the samples were stored at −20°C until analysis. The faecal samples were weighed after drying to a constant weight at 60°C in an oven and stored at −20°C until analysis.

Bile was collected through the bile duct, as described previously [22]. In brief, 5 rats were administered i.v. 50 mg/kg or i.g. 100 mg/kg through the same procedure described above. Biliary samples were collected at intervals of −1∼0 h predose and 0∼1, 1∼2, 2∼3, 2∼3, 3∼4, 4∼5 and 5∼6 h postdose, and the volume of each sample was recorded.

### Sample processing

One hundred microlitres of tissue homogenate and 20 µL of IS working solution (paracetamol, 250 ng/mL in methanol) was pipetted into a 1.5 mL polypropylene tube. Then, 280 µL of methanol was added, followed by 1 min of vortex mixing and 10 min of ultrasonic incubation. After centrifuging at 45,000 *g* for 5 min, the clear supernatant was transferred to a new tube and evaporated to dryness under a N_2_ stream. The residue was reconstituted in 200 µL of mobile phase solution.

To prepare urinary, faecal and biliary samples, a solid-phase extraction (SPE) purification step was performed after the sample preparation. After drying under a N_2_ stream, the residue was reconstituted in 1 mL ammonium acetate (pH 4, 20 mM) solution and carefully loaded on a SPE cartridge (Oasis HLB 60 mg; Waters), which was equilibrated with 3 mL methanol followed by 3 mL water before use. The cartridge was washed with water, followed by 1 mL methanol to elute salidroside and *p*-tyrosol. The eluate was evaporated to dryness under a N_2_ stream. The residue was reconstituted in 200 µL of the mobile phase and then analysed by LC-MS/MS.

When the concentration of salidroside or *p*-tyrosol in biosamples exceeded the linear calibration curve range, the biosamples were appropriately diluted with the blank solution before processing.

### Chromatographic and mass spectrometric conditions

The LC–MS/MS system consisted of an H-Class UPLC unit equipped with an ACQ UPLC BEH phenyl column (2.1 mm×50 mm, 1.7 µm), and an AB Sciex QTrap 5500 triple quadrupole mass spectrometer with an electrospray ionization source (Framingham, MA, USA) was used for the identification and quantification of salidroside and its metabolite, *p*-tyrosol. The column temperature was maintained at 30°C, and the auto-sampler temperature was set at 4°C. The optimised method used isocratic elution with a water (5 mM ammonium acetate)–acetonitrile mixture (9∶1, V/V) as the mobile phase at a flow rate of 0.25 mL/min. The total running time was 3 min. The injection volume was 5 µL. Paracetamol was used as the internal standard (IS).

ESI–MS/MS detection was performed in negative ion mode under the following conditions: ion spray voltage of −4500 V, source temperature of 600°C, nitrogen for nebuliser gas (gas 1) and turbo gas (gas 2) at 50 psi, nitrogen for curtain gas at 30 psi, entrance potential of −10 V, and dwell times of 80 ms. The transitions were set at m/z 299.1 → 119.0 for salidroside, m/z 136.9 → 119.0 for *p*-tyrosol and m/z 150.0 → 107.0 for the IS (**Table 1**). The data were acquired and processed using the analyst software version 1.6.1 (AB SCIEX).

**Table 1:** Mass spectrometric settings.

Compound	Q1 (*m/z)*	Q3(*m/z*)	CE(eV)	DP(V)	CXP(V)
Salidroside	299.1	119.0^a^	−18	−135	−28
	299.1	89.0	−19	−147	−12
*p*-Tyrosol	136.9	119.0^a^	−20	−108	−19
	136.9	106.0	−22	−105	−12
IS	150.0	107.0^a^	−24	−106	−12

a Ion for quantification.

### Preparation of Stock Solutions, Calibration Standard (CS) and Quality Control (QC) Samples

Stock solutions of salidroside, *p-*tyrosol and IS (paracetamol) were prepared at 200 µg/mL in acetonitrile-water (1∶9, v/v) and further diluted with acetonitrile-water (1∶9, v/v) to prepare standard solutions at selected concentrations. The 250 ng/mL IS working solution was diluted from a stock solution during use. All solutions were stored at −20°C until use.

The CSs and QCs were prepared by spiking 20 µL of the appropriate working solution with 100 µL of blank rat tissue homogenate (heart, liver, spleen, lung, kidney and brain), blank excretory samples (urine and bile) or 10 mg of blank faecal samples. Following sample processing, the spiked rat biosamples were treated using the same procedure as that used for the unknown samples.

### Method Validation

Method validation was performed for assay specificity, precision, linearity, accuracy, and extraction recovery under the LC-MS/MS analytical conditions described above. The specificity of the method was assessed by preparing and analysing six different batches of drug-free rat tissues. Each blank sample was processed through the proposed extraction procedure and tested to ensure no endogenous interference of the analyte from the rats. The assays of intra- or inter-day accuracy were performed in six separate runs on the same day or on six consecutive days, and expressed as (observed concentration/spiked concentration)×100%. The extraction recoveries of salidroside and *p-*tyrosol were determined at three QC levels, respectively. The matrix effects from endogenous substances present in extracted biosamples may cause ion suppression or enhancement of the signal. The matrix effects were investigated by post-extraction spike method in the present study [23]. The stability of the standard solutions was tested at room temperature for 4 h and upon refrigeration (4°C) for 30 days. The stability of the analytes was examined by keeping replicates of salidroside and *p*-tyrosol QC samples in the autosampler tray for 24 h and in a freezer at −80°C for 30 days; the freeze-thaw stability was obtained over three freeze-thaw cycles. For each concentration and each storage condition, 6 replicates were analysed in one analytical batch.

## Results

### Identification of salidroside and p-tyrosol in rat tissue and excretory samples

In different tissue and excretory samples, salidroside and *p*-tyrosol were identified by directly comparing with those standard compounds in MRM mode on an LC-MS/MS. The LC-MS/MS detection was performed in the negative ion mode, and the conditions for the detection of salidroside and *p-*tyrosol were optimised with the standards spiked in the tissue homogenates and excretory samples. The deprotonated molecular ion of salidroside ([M−H]-299.0) produced typical and characteristic fragment ions at *m/z* 118.8 and 179.0 with the structures of [*p-*tyrosol-OH]^−^ and glucosyl ions. The deprotonated *p-*tyrosol ([M−H]-137.0) produced daughter ions at *m/z* 105.8 (loss of a -CH_2_OH fragment) and 118.9 (loss of a -OH fragment) [19]. Salidroside and *p*-tyrosol were detected in all tested tissues, including liver, kidney, spleen, heart, lung and brain, and all excretory samples, i.e., faeces, urine and bile.

### Method Validation

**Figure 2:**
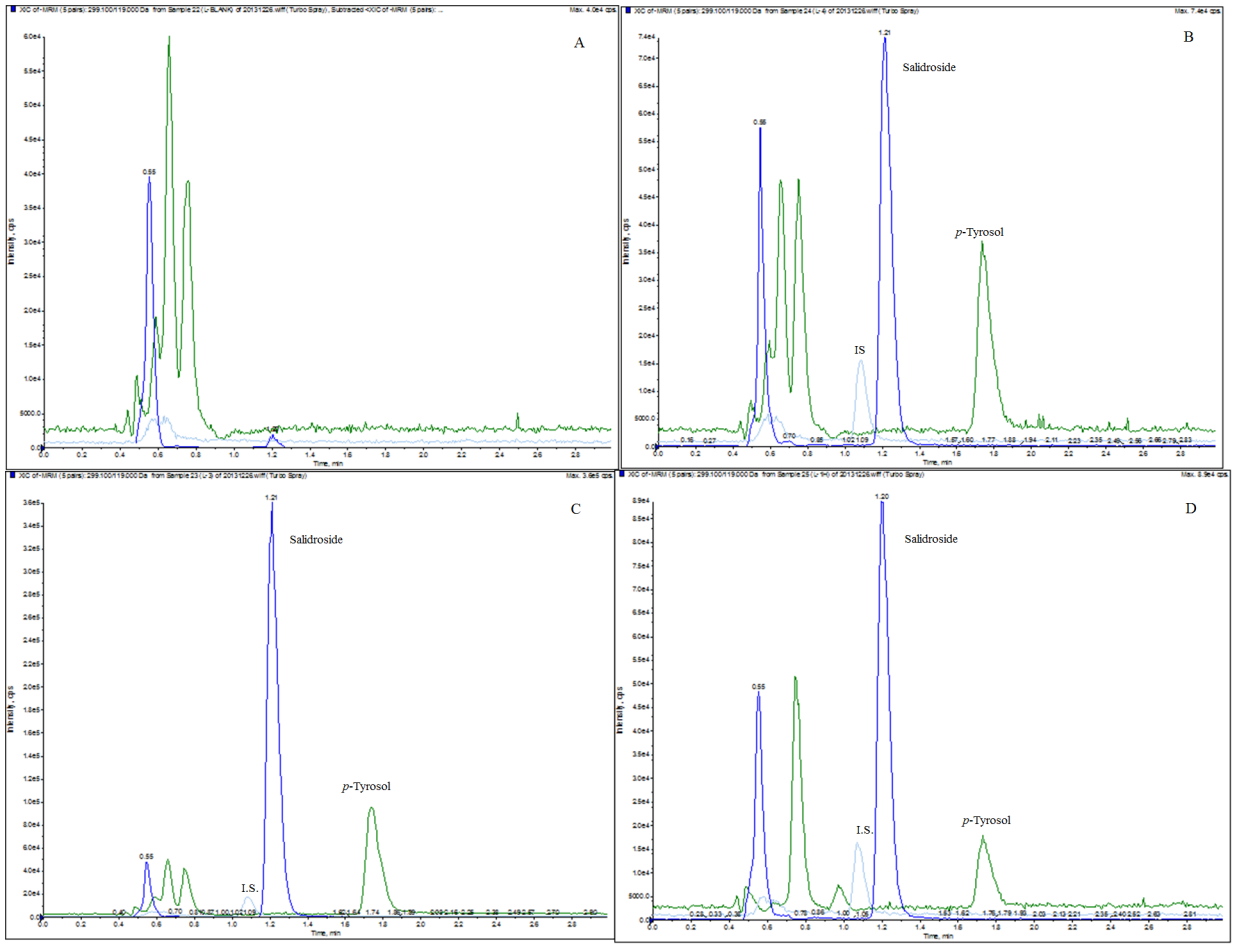
MRM chromatograms of salidroside, *p*-tyrosol and the IS in (A) a blank rat liver tissue homogenate sample, (B) a blank rat liver tissue sample spiked with salidroside (500 ng/mL), *p*-tyrosol (500 ng/mL) and the IS (200 ng/mL), (C) a rat liver tissue homogenate sample collected 0.17 h after i.v. administration of salidroside (50 mg/kg) with the IS (200 ng/mL), (D) a rat liver tissue homogenate sample collected 1 h after i.g. administration of salidroside (100 mg/kg) with the IS (200 ng/mL).

A quantification method for the simultaneous determination of salidroside and *p*-tyrosol in rat tissues and excretory samples by LC-MS/MS was developed and validated. **Figure 2** shows the representative chromatograms of the liver samples. The retention time was 1.21 min for salidroside, 1.74 min for *p*-tyrosol, and 1.08 min for the IS (paracetamol). No endogenous substance interfered with salidroside, *p*-tyrosol or the IS in any of the samples.

Salidroside and *p*-tyrosol were detected in all tested tissues and excretory samples, whereas salidroside could not be quantified in the lung, spleen and brain because the concentrations were below the LLOQ (50 ng/mL) in tissue homogenates. The regression equations, correlation coefficients, and linear ranges for the analytes analysed in different tissues and excretory samples are listed in **Table 2**. The correlation coefficients (*r^2^*) for the calibration curves were greater than 0.99, indicating good linearity over the concentration ranges for salidroside and *p*-tyrosol in all tissue and excretory samples analysed. When the concentration of the analytes exceeded the range of a calibration curve, appropriate dilutions were applied to the samples with blank biomatrices before sample processing.

**Table 2:** Calibration curves of salidroside and *p*-tyrsosol in biological matrices.

Tissues	compounds	Regression equation	Linear range (ng/mL)	Correlationcoefficient (*r^2^*)
Heart	salidroside	*Y* = 0.0006*x*+0.0013	20–200	0.9925
	*p*-tyrosol	*Y* = 0.0011*x*+0.0482	50–2000	0.9902
Liver	salidroside	*Y* = 0.0002*x*+0.0137	50–2000	0.9959
	*p*-tyrosol	*Y* = 0.0004*x*–0.0022	50–2000	0.9951
Spleen	salidroside	–	–	–
	*p*-tyrosol	*Y* = 0.0023*x*+0.0522	50–2000	0.9997
Lung	salidroside	–	–	–
	*p*-tyrosol	*Y* = 0.0035*x*–0.1022	50–2000	0.9902
Kidney	salidroside	*Y* = 0.0002*x*+0.012	50–2000	0.9973
	*p*-tyrosol	*Y* = 0.0010*x*+0.0658	50–2000	0.9933
Brain	salidroside	–	–	–
	*p*-tyrosol	*Y* = 0.0310*x*–0.0134	50–2000	0.9920
Faeces	salidroside	*Y* = 0.0005*x*+0.0925	50–2000	0.9924
	*p*-tyrosol	*Y* = 0.0007*x*+0.2106	50–2000	0.9939
Urine	salidroside	*Y* = 0.0012*x*–0.0176	50–2000	0.9936
	*p*-tyrosol	*Y* = 0.0031*x*–0.0459	50–2000	0.9949
Bile	salidroside	*Y* = 0.0002*x*+0.0166	50–2000	0.9981
	*p*-tyrosol	*Y* = 0.0010*x*+0.0658	20–200	0.9933

*Y*: peak area ratios of analytes to the IS; *x*: concentrations of analytes in matrices.

- not applied for the concentrations of analytes were bellow LLOQ.

The intra- and inter-day precision and accuracy for salidroside and *p-*tyrosol were evaluated by assaying the QC samples for six consecutive days. The data suggested that the method was accurate and precise for the simultaneous analysis of salidroside and *p*-tyrosol in rat tissue samples. The recoveries of salidroside and *p-*tyrosol spiked into rat samples were determined at three QC levels. The relative extraction recoveries were 89.04%∼114.19% for salidroside and *p*-tyrosol, which indicated that the method was reproducible and reliable. The matrix effects ranged from 82.47 to 112.63% for salidroside and from 82.72 to 115.60% for *p-*tyrosol, and the RSD was less than 15.0%, which indicated that ion suppression or enhancement from matrices could be treated as negligible in this study (**Table 3** and **4**).

**Table 3:** Accuracy, precision, recovery and matrix effects for the determination of salidroside in different matrixes (n = 6).

Analyte	Concentration (ng/mL)	Intra-day			Inter-day			Recovery		Matrix effects	
		Mean±SD (ng/mL)	Precision (%)	Accuracy (%)	Mean±SD (ng/mL)	Precision (%)	Accuracy (%)	Mean±SD	RSD (%)	Mean±SD	RSD (%)
Heart	20	22.02±1.78	8.10	110.12	22.40±1.87	8.35	111.98	110.39±11.45	10.37	91.56±7.06	7.71
	100	103.10±11.21	10.87	103.10	107.84±5.36	4.97	107.84	98.81±13.39	13.55	106.84±16.99	15.91
	200	205.76±21.94	10.66	102.88	204.32±25.90	12.68	102.16	98.29±11.25	11.45	89.99±2.01	2.24
Liver	50	52.45±6.71	12.79	104.90	51.26±5.11	9.97	102.51	111.44±10.26	9.20	92.45±3.27	3.54
	500	550.51±28.53	5.18	110.10	563.64±19.79	3.51	112.73	108.10±3.48	3.22	83.845±3.61	4.32
	2000	2126.53±235.05	11.05	106.33	2121.73±302.85	14.27	106.09	102.00±9.03	8.85	87.63±6.77	7.72
kidney	50	48.23±6.74	13.97	96.46	48.12±5.32	13.96	96.24	92.15±10.37	11.25	112.63±7.96	7.06
	500	535.58±52.86	9.87	107.12	552.35±17.58	3.18	110.47	106.23±14.85	13.98	112.37±11.44	10.18
	2000	2133.40±128.80	6.04	106.67	2017.59±140.74	6.68	105.40	110.16±5.87	5.33	95.33±8.80	9.23
Urine	50	51.75±1.15	2.23	103.5	51.29±1.57	3.07	102.57	103.57±2.82	2.73	98.43±10.14	10.30
	500	509.91±53.62	10.52	101.98	510.34±61.99	2.20	102.07	102.61±13.04	12.71	111.60±25.42	22.77
	2000	1950.44±90.48	4.64	97.52	1888.79±76.24	4.04	94.44	95.79±3.57	3.73	84.90±3.06	3.61
Faeces	50	47.97±4.86	10.13	95.95	44.08±0.84	2.00	88.17	91.90±6.58	7.16	93.46±13.32	14.25
	500	503.99±48.99	9.72	100.80	504.66±73.47	14.56	100.93	98.84±11.00	11.13	82.47±6.57	7.97
	2000	2057.01±182.45	8.87	102.85	1923.64±99.44	5.17	96.18	99.01±6.03	6.09	87.78±3.07	3.50
Bile	50	50.81±6.29	12.38	101.63	49.41±1.49	3.02	98.82	101.08±15.35	15.18	88.80±6.97	7.85
	500	520.79±42.14	8.09	104.16	556.20±14.66	2.64	111.24	106.99±7.66	7.16	88.82±2.15	2.42
	2000	2057.50±101.50	4.93	102.88	2022.65±47.12	2.33	101.13	100.47±2.02	2.01	92.34±6.87	7.44

**Table 4:** Accuracy, precision, recovery and matrix effects for the determination of *p*-tyrosol in different matrixes (n = 6).

Analyte	Concentration(ng/mL)	Intra-day			Inter-day			Recovery		Matrix effects	
		Mean±SD (ng/mL)	Precision (%)	Accuracy (%)	Mean±SD (ng/mL)	Precision (%)	Accuracy (%)	Mean±SD	RSD (%)	Mean±SD	RSD (%)
Heart	50	50.95±5.25	10.30	101.90	48.51±3.90	8.04	97.03	102.82±14.73	14.32	90.98±9.79	10.76
	500	496.62±37.18	7.49	99.32	516.32±35.97	6.97	103.26	102.93±7.76	7.54	101.16±9.21	9.11
	2000	2046.91±215.38	10.52	102.35	2950.35±1170.56	6.03	97.52	103.93±14.89	14.33	89.88±3.89	4.33
Liver	50	49.16±5.13	10.44	98.33	46.19±3.85	8.54	92.38	105.44±1.99	1.88	85.61±8.71	10.17
	500	481.62±31.32	6.80	96.32	467.05±30.99	6.64	93.41	105.50±3.63	3.44	87.13±1.78	2.04
	2000	1991.17±50.72	2.55	99.56	2011.83±12.92	0.64	100.59	113.04±15.43	13.65	92.49±3.27	3.53
Spleen	50	54.57±5.88	10.78	109.14	48.96±6.31	12.89	97.93	97.93±12.62	12.89	89.07±15.18	17.04
	500	469.79±56.20	11.96	93.96	445. 19±62.74	14. 09	89.04	89.04±12.55	14.09	91.25±4.23	4.63
	2000	2059.49±276.27	13.41	102.97	1889.66.59±170.54	9.03	94.48	94.48±8.53	9.03	94.80±5.88	6.20
Lung	50	52.37±4.40	8.41	104.73	50.13±1.74	3.47	100.27	97.98±2.74	2.80	90.88±9.39	11.61
	500	510.24±50.35	9.87	102.70	553. 41±11. 00	1. 99	110.68	99.15±11.34	11.44	114.72±14.60	10.33
	2000	2032.63±189.52	8.87	106.63	2073. 51±76.06	3.67	103.68	99.69±6.40	6.42	101.75±6.80	6.68
kidney	50	47.20±5.65	11.97	94.40	49.28±3.24	6.59	98.55	98.55±6.47	6.86	112.83±16.16	14.32
	500	516.96±54.33	10.51	103.39	491. 53±23.65	4. 81	98.31	98.31±4.73	4.81	90.07±3.99	4.42
	2000	2016.55±278.20	13.80	100.83	2017.59±140.74	6.68	105.40	100.83±13.91	13.80	83.371±11.31	13.51
Brain	50	55.42±3.63	6.55	110.84	56.24±1.28	2.28	112.49	114.19±3.46	3.03	82.72±2.50	3.02
	500	511.34±60.19	11.77	102.27	555. 59±54.78	9. 86	111.12	105.49±12.45	11.81	115.60±12.28	10.62
	2000	2020.71±142.69	7.06	101.04	2114.32±141. 42	6.69	105.72	105.93±5.01	4.73	101.57±1.96	1.93
Urine	50	54.18±4.96	9.15	108.37	56.22±1.51	2.69	112.44	112.25±7.57	6.75	85.06±15.06	17.70
	500	487.52±30.93	6.34	97.50	513. 81±4.31	0. 84	102.38	99.70±5.34	5.36	92.95±3.75	4.04
	2000	1998.81±78.17	3.91	99.94	2027.51±108. 47	5.35	101.38	99.74±4.76	4.77	93.57±8.09	8.65
Faeces	50	55.42±3.63	6.55	110.84	53.78±6.36	11.83	107.56	105.85±9.47	8.95	91.32±3.01	3.29
	500	511.34±60.19	11.77	102.27	507. 35±22.97	4. 53	101.47	100.81±3.45	3.42	91.90±3.18	3.46
	2000	2020.71±142.69	7.06	101.04	1929.98±29. 91	1.55	96.50	96.38±1.08	1.17	95.09±2.04	2.15
Bile	20	20.87±1.51	7.21	104.36	19.93±1. 27	6.38	99.66	104.30±9.22	8.84	109.01±24.35	22.34
	100	94.24±9.72	10.32	94.24	99.39±13.03	13.11	99.39	95.31±11.61	12.18	91.39±7.34	8.07
	200	199.43±5.56	2.79	99.72	200.01±1.72	0.86	100.01	98.70±2.33	2.37	106.50±9.39	8.81

After being placed in the auto-sampler at room temperature for 24 h, the concentrations of analytes in different matrices deviated less than ±15% from their nominal concentrations. In the freeze-thaw and long-term stability tests, the concentrations obtained were higher than 85% of their nominal concentrations. The data suggested no significant analyte loss during sample storage and processing.

### Tissue distribution studies

**Figure 3:**
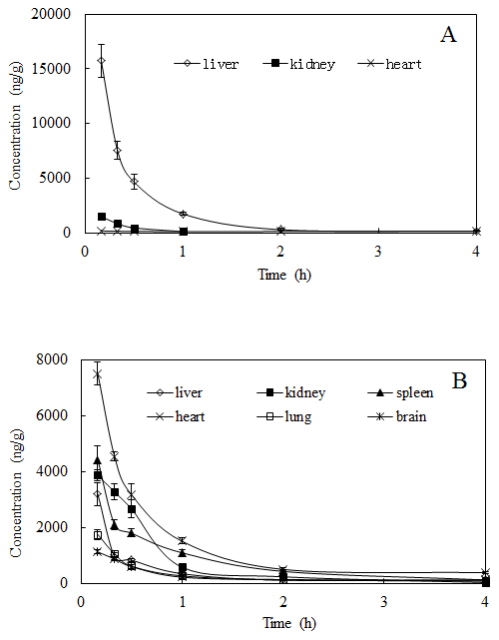
Mean concentration-time profiles of (A) salidroside and (B) *p-*tyrosol in rat tissues (n = 6) obtained after i.v. administration of salidroside (i.v. 50 mg/kg).

**Figure 4:**
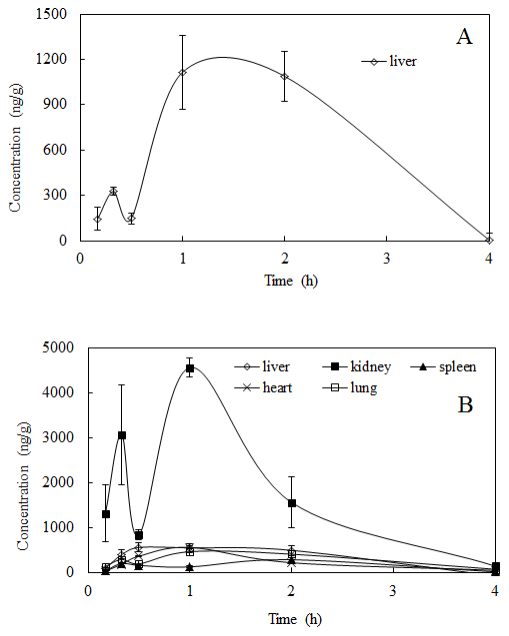
Mean concentration-time profiles of (A) salidroside and (B) *p-*tyrosol in rat tissues (n = 6) obtained after i.g. administration of salidroside (i.g. 100 mg/kg).

To investigate the distribution of salidroside and *p-*tyrosol in rats after the i.v. administration at a dose of 50 mg/kg and the i.g. administration at a dose of 100 mg/kg salidroside to rats, the concentrations were determined in tissues within 4 h after administration, and the tissue concentration–time profiles are shown in **Figure 3** and **Figure 4**, respectively.

After the i.v. administration of salidroside to rats, salidroside was detected in all tested tissues (liver, kidney, heart, lung, spleen and brain); however, salidroside could be quantified only in the liver, kidney, and heart tissues (**Figure 3A**). The maximum levels of salidroside observed were 15713.30±2987.87 ng/g in the liver, 1480.54±324.69 ng/g in the kidney and 170.34±20.60 ng/g in the heart. Salidroside was eliminated rapidly from tissues, and the concentrations were very low with no significant difference in the tissues after 2 h of administration. Salidroside was also detected in the spleen, lung and brain, based on S/N = 3; however, the concentrations could not be measured because they were below the LLOQ in the tissue homogenates (50 ng/mL).

Salidroside was extensively metabolised to its aglycone in the tissues after i.v. administration, and the results (**Figure 3B**) indicated that *p*-tyrosol underwent a rapid and wide distribution in the tissues throughout the entire body within 4 h of i.v. administration. The maximum concentrations were observed at the first time point, 0.17 h, which implied that the maximum concentration could be higher and may be achieved earlier than the time point used here. The maximum levels of *p*-tyrosol were detected in the heart (7497.17±103.87 ng/g), followed by the spleen (4421.92±962.32 ng/g), kidney (3857.97±414.92 ng/g), liver (3190.49±832.98 ng/g) and lungs (1742.90±348.68 ng/g), in order. Additionally, *p*-tyrosol showed a substantial distribution (1118.38±158.14 ng/g) in the brain. After 0.17 h of administration, the concentrations of *p*-tyrosol declined abruptly and were almost non-detectable after 4 h of administration.

After a single i.g. administration of 100 mg/kg salidroside, salidroside was detected only in the liver. Only a small amount of salidroside was found within the first 0.5 h, which increased gradually; the maximum concentrations of 1113.07±244.31 ng/g and 1088.87±165.66 ng/g occurred at the 1 h and 2 h time points after administration, respectively, and decreased thereafter (**Figure 4A**).

In contrast, *p-*tyrosol was detectable in most tissues except the brain after i.g. administration, and the kidney tissues showed a significant amount of *p-*tyrosol compared with the other tissues. The levels of *p-*tyrosol in the kidney were much higher than those in the other tissues, which reached 3075.56±1110.50 ng/g at 0.33 h and 4568.00±207.86 ng/g at 1 h after administration. Among the other tissues, the level of *p-*tyrosol in the liver was higher, followed by the heart at 0.5 h after administration, and the level in the spleen was significantly lower than the others at 1 h postdose. At the other time points, the *p-*tyrosol contents were not significantly different among the tissues (**Figure 4B**).

### Excretion studies

The excretion of salidroside and its aglycone metabolite *p*-tyrosol in the urinary, faecal, and biliary samples are summarised in **Table 5** **,** **Table 6** and **Table 7**, respectively.

**Table 5:** Cumulative content of salidroside and *p*-tyrosol in urinary samples following i.v. and i.g. administration of salidroside (i.v. 50 mg/kg, i.g. 100 mg/kg) (n = 6).

Time (h)	i.v. administration (µg)		i.g. administration (µg)	
	Salidroside	*p*-tyrosol	Salidroside	*p*-tyrosol
0–12	6704.77±494.55	2.60±0.14	3698.97±649.45	3.64±0.08
12–24	501.45±65.03	5.60±0.90	638.13±117.12	22.04±0.75
24–48	521.55±65.83	2.39±0.35	Nd	327.25±121.05
48–72	272.27±20.78	0.27±0.03	Nd	85.06±19.11
0–72	8000.06±519.38	10.86±0.43	4337.10±766.43	407.10±37.08
Excretion (% of dose)	64.00±4.15	0.19±0.01	23.80±3.32	2.25±0.28

Nd: not detectable.

**Table 6:** Cumulative content of salidroside and *p*-tyrosol in faecal samples following i.v. and i.g. administration of salidroside (i.v. 50 mg·kg^−1^, i.g. 100 mg·kg^−1^) (n = 6).

Time (h)	i.v. administration (µg)		i.g. administration (µg)	
	Salidroside	*p*-tyrosol	Salidroside	i.g.
0–12	2.73±0.51	13.79±0.71	Nd	0.01±0.00
12–24	13.90±1.60	10.73±1.75	Nd	0.03±0.01
24–48	19.34±1.51	50.60±2.73	Nd	0.04±0.02
48–72	1.06±0.07	9.71±0.87	Nd	0.03±0.00
0–72	37.04±1.06	85.03±2.21	Nd	0.13±0.03
Excretion (% of dose)	0.30±0.01	1.48±0.04	Nd	0.00±0.00

Nd: not detectable.

**Table 7:** Cumulative content of salidroside and *p*-tyrosol in biliary samples following i.v. and i.g. administration of salidroside (i.v. 50 mg/kg, i.g. 100 mg/kg) (n = 6).

Time (h)	i.v. administration (µg)		i.g. administration (µg)	
	Salidroside	*p*-tyrosol	Salidroside	*p*-tyrosol
0–1	281.00±9.39	0.55±0.05	0.14±0.03	0.04±0.00
1–2	52.02±4.27	0.55±0.02	0.22±0.03	0.04±0.01
2–3	9.90±0.12	0.05±0.00	0.74±0.06	0.05±0.00
3–4	5.80±0.44	0.05±0.01	1.18±0.19	0.05±0.00
4–5	5.62±0.12	0.06±0.00	1.79±0.07	0.06±0.01
5–6	4.42±0.38	0.05±0.00	0.53±0.09	0.07±0.01
0–6	358.77±5.54	1.30±0.04	4.65±0.13	0.32±0.02
Excretion (% of dose)	2.86±0.04	0.02±0.00	0.02±0.00	0.00±0.00

Salidroside was excreted rapidly, mainly in the urine, after a single-dose i.v. administration of 50 mg/kg and i.g. administration of 100 mg/kg salidroside. After the i.v. administration, the cumulative excretion of salidroside reached 6704.77±494.55 µg within 12 h, and the total cumulative amounts were 8000.06±519.38 µg after 72 h, which represented 64.00±4.15% of the administered salidroside. After i.g. administration, the cumulative excretion of salidroside reached 3698.97±649.45 µg within 12 h, the total cumulative amounts were 4337.10±766.43 µg after 72 h, which represented 23.80±3.32% of the administered salidroside. In the urine, *p*-tyrosol could be detected until 72 h after dosing, and the cumulative amounts were 10.86±0.43 µg (0.19±0.01% of dose) and 407.10±37.08 µg (2.25±0.28% of dose) 72 h after the i.v. and i.g. administrations.

After the i.v. administration of 50 mg/kg salidroside, the total cumulative amounts of salidroside and *p*-tyrosol in faeces reached 37.04±1.06 µg and 85.03±2.21 µg after 72 h, which represent 0.30±0.01% and 1.48±0.04% of the administrated amount, respectively. In contrast, after i.g. administration, salidroside was undetectable and only a trace amount of *p*-tyrosol was detected in faeces.

After the i.v. administration, salidroside and *p*-tyrosol were detectable up to 6 h in the bile and the cumulative amounts were 358.77±5.54 µg, and 1.30±0.04 µg which represented 2.86±0.04% and 0.02±0.00% of the i.v. administered salidroside, respectively. Whereas after i.g. administration, only 0.02±0.00% of the administered dose (4.65±0.13 µg) of salidroside and 0.00±0.00% (0.32±0.02 µg) of *p*-tyrosol were detected 6 h of post-administration.

## Discussion

During their transport across the jejunum and ileum, phenol glycosides are subjected to extensive metabolism by phase I hydrolysing and oxidising enzymes, including cytochrome P450 and glucosidase enzymes and by phase II conjugating and detoxifying enzymes. The deglycosylation by β-glucosidases present in cell-free extracts of the human small intestine and liver, which can hydrolyse various phenolic glycosides, is a critical step in the absorption and metabolism of dietary phenol glycosides [2], [24], [25]. The majority of aglycones, which escape metabolism in the small intestine, are metabolised by phase I and II enzymes, including uridine diphosphate (UDP)-glucuronosyl transferases, which lead to the formation of *O*-glucuronides. Sulpho-transferases generate *O*-sulphates and catechol-*O*-methyl transferases, which produce *O*-methylated metabolites [26], [27], [28], [29], [30], and the aglycones may be negligibly present in the circulation [31]. In our previous study, *p*-tyrosol was identified as the deglycosylation metabolite of salidroside in the plasma after i.v. administration to rats at a dose of 50 mg/kg; however, it was not detectable after i.g. administration through HPLC-PDA and LC-MS/MS analysis [19]. Based on reports from the literature, we hypothesised that salidroside may metabolise to *p*-tyrosol after i.g. administration, but it may be further metabolised to its sulphate or glucuronide conjugates, or even the methylate, which would also result in a lack of detectable *p*-tyrosol in the plasma sample [24].

In the present study, the metabolism of salidroside to its aglycone *p*-tyrosol was determined after i.v. and i.g. administrations.

In testing tissue homogenate samples, including the liver, kidney, spleen, heart, lung and brain, the highest level of salidroside was found in the liver, followed by the kidney and heart, and it was detected but could not be quantified in the spleen, lung, and brain. *p-*Tyrosol could be observed in all tissues at 0.17 h after the i.v. administration of salidroside (50 mg/kg), which decreased rapidly and could not be detected after 4 h of administration. The results of the current study indicate that after i.v. administration, salidroside was extensively metabolised to *p*-tyrosol, then distributed to various organs and cleared rapidly. The highest levels of *p*-tyrosol were detected in the heart, followed by the spleen, kidney, liver and lungs, in that order. Additionally, *p*-tyrosol showed substantial disposition in the brain. The maximal concentrations of salidroside and *p*-tyrosol in the tissues were found at 0.17 h after dosing, indicating that their distribution rate from blood into tissues was extremely rapid after i.v. administration.

Salidroside was found only in the liver but not in the other tested tissues (kidney, heart, spleen, lung and brain), and *p-*tyrosol was detectable in all tissues except in the brain after the i.g. administration of salidroside to rats. The kidneys contained a significantly greater amount of *p-*tyrosol than other tissues. *p*-Tyrsosol was widely distributed in rat tissues after both types of administration, which indicates its ready hydrolysis from salidroside to *p*-tyrosol *in vivo*.

The contents of salidroside and *p*-tyrosol found in the tissues after i.g. administration (100 mg/kg) in rats were significantly lower than those found after i.v. administration (50 mg/kg). Altogether, these data suggest that only a small amount of the compound can be dissolved in the gastrointestinal fluid to be absorbed into the bloodstream when administered by the oral route. In fact, the majority of orally administered drugs are absorbed into the systemic circulation via the portal blood and undergo first pass metabolism, thus exhibiting low oral bioavailability [32]. It has been reported that salidroside has various biological activities, including a hepatoprotective effect and protective effects against cardiomyocyte death and anoxia/reoxygenation damages on myocardium [9], [10]. The high accumulation of salidroside in the liver was in accordance with its hepatoprotective function. Higher *p-*tyrosol, but not salidroside, levels were detected in the heart, especially after i.g. administration. Salidroside could not be detected in most tissues, which may imply that *p-*tyrosol is the active agent accounting for the bioactivity of salidroside. *p-*Tyrosol has also been reported to penetrate and accumulate in macrophages and improve the intracellular antioxidant defence systems, as well as to counteract cardiovascular diseases [11].

Our results indicate that urinary elimination is the major route for the excretion of salidroside. After i.v. administration, 64.00±4.15% of the total dose was excreted in the form of salidroside through urine in rats whereas, 23.80±3.32% of the administered salidroside was eliminated via urine after i.g. administration. In addition, 0.19±0.01% and 2.25±0.28% of the dose were excreted in the form of *p-*tyrosol through urine after the i.v. and i.g. administration, respectively. The excretion level of salidroside was significantly higher than that of *p*-tyrosol in rat urine, possibly because different structures present different lipophilic properties.

The salidroside and *p*-tyrosol in the faeces constituted 0.3±0.01% and 1.48±0.04% of the total dose after i.v. administration, respectively. The results clearly show that salidroside was metabolised or degraded through intestinal enzyme activity and the activity of microbial bacteria after being excreted into the intestine via the biliary system. After the i.g. administration of salidroside, the total recoveries of both salidroside and *p*-tyrosol in the faeces within 72 h were approximately 0%. In addition, the biliary excretion levels after i.v. and i.g. administration were 2.86±0.04% and 0.02±0.00% of the dose, respectively.

The total excretion rates for salidroside as both salidroside and *p*-tyrosol were 68.85% and 26.07% after i.v. and i.g. administration, respectively. This result indicates that salidroside underwent more extensive metabolism after i.g. administration than after i.v. administration, similar to flavonoid glycosides, and is metabolised by phase I or phase II enzymes once absorbed. The first stage of metabolism is likely to be deglycosylation by cytosolic β-glucosidase, and cytochrome P450 mono-oxygenase-dependent activities that may be involved include hydroxylation and demethylation. The cytochrome mono-oxygenase enzymes, methyltransferase and conjugating enzymes may contribute to the conjugation metabolism [24], [25]. After i.g. administration, salidroside and its metabolite *p*-tyrosol might be further metabolised to its sulphate or glucuronide conjugates, or even the methylate, resulting in its low recovery in the forms of salidroside and its aglycone *p*-tyrosol in the excretory samples. Thus, more attention will be paid to the further metabolism and metabolites of salidroside in our further studies. Normally, further metabolism after deglycosylation is part of self-detoxification by an organism to destroy xenobiotics and protect itself. Hence, further metabolism results in decreased absorption and lower activity. Improved efficiency and therapeutic function of salidroside would be achieved by i.v. administration. Although i.g. administration preserves the original form in the circulatory system and *p*-tyrosol in the active form is distributed in the organs, oral administration is more convenient and acceptable for health-food products.

## Conclusions

In the present study, the metabolism of salidroside to its aglycone *p*-tyrosol was determined after i.v. and i.g. administrations. After i.v. administration, salidroside was extensively metabolised to *p*-tyrosol, then distributed to various organs and cleared rapidly. The highest levels of *p*-tyrosol were detected in the heart, followed by the spleen, kidney, liver and lungs, in that order. Additionally, *p*-tyrosol showed substantial disposition in the brain. Salidroside was found only in the liver, but most of the analysed tissues contained *p-*tyrosol, except the brain after the i.g. administration of salidroside to rats, and the kidney contained a significantly greater amount of *p-*tyrosol compared with the other tissues. Urinary elimination is the major route for the excretion of salidroside after i.v. and i.g. administration and salidroside underwent more extensive metabolism after i.g. administration than after i.v. administration. The information included in this study may be very useful to advance the knowledge regarding salidroside bioactivity and beneficial health effects.

## Supporting Information

Checklist S1 **ARRIVE Guidelines Checklist.**
(DOC)
